# Comment on “Occupational Exposure to Blood and Body Fluids in a Department of Oral Sciences: Results of a Thirteen-Year Surveillance Study”

**DOI:** 10.1155/2014/906585

**Published:** 2014-03-19

**Authors:** Virginia Di Bari, Gabriella De Carli, Vincenzo Puro

**Affiliations:** Emerging and Re-Emerging Infections Unit and AIDS Referral Centre, Department of Epidemiology and Pre-Clinical Research, National Institute for Infectious Diseases “Lazzaro Spallanzani,” IRCCS, 00149 Rome, Italy

Gatto and coworkers analyze dental care workers (DCW) injuries which occurred in the Department of Oral Sciences of the University of Bologna over a 13-year period, with the aim of assessing if additional safety precautions or modification of current procedures are needed.

Their findings show that the device more frequently involved in accidents is the needle for local anaesthesia (41% of percutaneous injuries), disposable and nondisposable, and that students are those most frequently exposed (40%), especially during instruments reprocessing and disposal [1]. The authors conclude that “an adequate prevention could have avoided only eye injuries.” As our research group on occupational risk of infection with bloodborne pathogens (Studio Italiano Rischio Occupazionale da HIV e da altri patogeni a trasmissione ematica, SIROH) has collaborated in the development of the European Directive 2010/32/EU protecting healthcare workers from needle and sharps injuries [2], which will integrate Italian legislation on occupational risk in healthcare, we support additional safety precautions—included in the directive—for the prevention of occupational injuries in DCW.

Indeed, in our opinion, in their analysis they fail to consider (1) including modifications in the protocol for using and disposing of carpules, for example, through a wider adoption of disposable devices, instead of using nondisposable syringes, which clearly need additional manipulation for reprocessing, and (2) the implementation of devices incorporating a safety-engineered mechanism (safety-engineered devices (SED)), while available, within the possible preventive interventions.

Both measures appear within the requirements of the European Directive, which supports an overall preventative strategy including safer procedures for the use and disposal of used sharps and the provision of SED where there is a risk of injury and contact with the patient's blood.

A study in the United Kingdom (UK) is a good example of both measures [3]. The authors describe the steps in the introduction of a safety syringe replacing a nondisposable device into a UK dental school. These steps include, among others, the collection of evidence for the need for a change, the training of staff, and the feedback to the manufacturer on the new device. As for the need for a change, they considered all avoidable needlestick injuries which had occurred in their facilities over a 3-year period and identified those occurring at a dedicated clinic dealing exclusively with patients with bloodborne viruses (predominantly HIV) due to the use of nondisposable syringes and those occurring among staff at a dental school during recapping and dismantling of the syringes, or while the needle was lying uncovered in an operating area. All these were considered preventable by the use of appropriate safety syringes.

SED for local anesthesia are available on the market, and they have been adopted with different levels of satisfaction depending on the device used [4, 5]. In the UK experience reported above, the Oral Medicine Department evaluated four different devices, two completely disposable and two where a component remains: after an improvement in design resulting from talks with the manufacturer, they eventually introduced a syringe with a wide sheath to protect needle when not in use, a nondisposable handle which however does not require autoclaving unless contaminated with blood, and a cartridge which is disposed with needle, quickly and easily, and can be replaced if necessary. Moderately intensive training was required at the introduction: all grades of staff as well as all dental students were included, and trainer nurses and training videos were used by the school to ensure further training of new staff.

In the second year of use of the safety syringes, a marked reduction in the incidence of avoidable needlestick injuries was detected (from 11.8 to 0 injuries per 1,000,000 hours worked per year and from 20.5 to 0 per 1,000 employees), in comparison to a control unit in the same school, undergoing the same training but using nondisposable syringes (from 26 to 20 injuries per 1,000,000 hours worked per year and from 45.2 to 33.9 per 1,000 employees). According to the authors, the reduction of injuries in the control unit, although not as marked as the one observed in the study unit, demonstrates the value of the programs for education and awareness raising [3].

Finally, implementing additional preventive measures is of particular importance as the reported injuries could be only the tip of the iceberg. Indeed, the overall number of injuries described by Gatto and coworkers could be significantly underestimated; the high percentage of bloodborne-infected patients among the sources of these injuries (22%) could be also the result of an overreporting of injuries involving patients with known or suspected infection. In the literature, injury rates among DCW and students vary greatly, depending on the system used to detect exposures (active observation, passive surveillance of reported injuries, and recalled injuries in surveys): data collected during the last 3 years of the postexposure surveillance program at the New York University College of Dentistry, which had a rate per 10,000 visits similar to that reported by Gatto and coworkers, showed a rate for students of 4/100 person-years and a rate for the faculty and staff of 2/100 person-years [6], while a recent survey carried out among German DCW and students identified an exposure rate of 43 and 74 injuries per 100 person-years, respectively [7].

We therefore suggest considering the local implementation of the measures indicated in the Directive 2010/32/UE to provide a safer working environment. This is of special importance in medical schools, where personnel in training is more vulnerable to injuries.
